# Structural and mechanistic insights into *α*2*β*1 and *α*5*β*1 integrin targeting by bioengineered extracellular vesicles originating from lung cancer cells

**DOI:** 10.1038/s41598-026-46071-2

**Published:** 2026-03-27

**Authors:** Anna M. Nowicka, Teresa Żołek, Agata Kowalczyk, Ireneusz P. Grudzinski

**Affiliations:** 1 Faculty of Chemistry, University of Warsaw, 1 Pasteura St, Warsaw, PL-02-093 Poland; 2 Department of Organic and Physical Chemistry, Faculty of Pharmacy, Medical University of Warsaw, 1 Banacha St, Warsaw, PL-02-097 Poland; 3 Department of Toxicology and Food Science, Faculty of Pharmacy, Medical University of Warsaw, 1 Banacha St, Warsaw, PL-02-097 Poland

**Keywords:** Integrins *α*2*β*1 and *α*5*β*1, Lung cancer–derived extracellular vesicles, Molecular dynamics simulations, Ligand-induced conformational changes in integrins, Binding affinity of the PTHTRWA-EVs-integrin complex, Biochemistry, Biophysics, Cancer, Cell biology

## Abstract

Integrins are transmembrane receptors that mediate bidirectional signaling across the plasma membrane and play a crucial role in tumor progression, metastasis, and cellular communication. In this study, we performed a comparative structural and biophysical analysis of the PTHTRWA-functionalized extracellular vesicles (PTHTRWA-EVs) interacting with *α*2*β*1 and *α*5*β*1 integrins to investigate the molecular determinants underlying selective recognition. Surface plasmon resonance experiments were used to characterize multivalent bioengineered EVs --- integrin binding under physiological conditions, while molecular dynamics simulations provided residue-level insight into local ligand-receptor interaction patterns and conformational preferences. These results indicate that PTHTRWA binding is associated with local conformational rearrangements consistent with stabilization of an open-like binding geometry at the integrin interface. Together, these complementary approaches highlight the potential of PTHTRWA-functionalized EVs as a platform for targeted drug delivery and cancer diagnostics.

**Supplementary Information:**

The online version contains supplementary material available at 10.1038/s41598-026-46071-2.

## Introduction

Over the past two decades, lung cancer has consistently ranked among the three most frequently diagnosed malignancies worldwide^1,2^. Unfortunately, the course of this disease is highly insidious - it develops without symptoms for a long time, meaning it is often diagnosed too late to be treated effectively^3^. These factors highlight the urgent need for new, more precise, and less toxic therapeutic strategies. Undoubtedly, the key element of effective lung cancer therapy is individualized treatment^4^. The choice of the best treatment strategy should take into account the specific characteristics of the tumor (subtype, stage, and location), as well as the patient’s health, comorbidities, and preferences^5^. In recent years, there has been a particular intensification of research into targeted therapy^6–10^. It is a modern method of cancer treatment that involves identifying and attacking specific characteristics of cancer cells. It works by inhibiting specific molecular pathways, which allows for the precise targeting of only those cells that are responsible for the development of cancer^11^. This therapy is often referred to as personalized medicine it considers the individual characteristics of each patient’s cancer. Thanks to research into the mechanisms of cancer development, targeted therapy has become a key element of modern cancer treatment. Research in this area includes both the search for new forms of drug administration and improvements in delivering the drug only to cancer cells.

In the context of precise drug delivery systems targeting cancer cells, extracellular vesicles (EVs) have attracted particular attention in recent years. The main focus of research is on the use of extracellular vesicles as natural, biodegradable nanovesicles for transporting drugs directly to cancer cells^12,13^. Their natural ability to communicate between cells and the fact that EVs are secreted by most eukaryotic cells, and are therefore unique to each organism, like fingerprints, allows them to potentially bypass the drug resistance mechanisms of tumors^14,15^. Unlike synthetic drug carriers such as liposomes, polymers, dendrimers, or micelles^16–18^, extracellular vesicles are largely non-toxic, capable of efficiently penetrating cell membranes, and can be engineered for targeted delivery^19^. This modification involves introducing a navigating ligand to the surface of EVs, such as transferrin, folic acid, aptamers, or short peptides^20^. This type of targeted therapy can significantly increase the effectiveness of treatment while reducing the harmful side effects associated with traditional methods. In addition, analysis of the molecular profile of a patient’s extracellular vesicles can provide real-time information about the biology of the tumor and help in selecting the most effective therapy^21^.

According to the literature the heptapeptide PTHTRWA can be successfully applied in the role of navigation ligand^22^. The authors’ studies using an in vitro cellular binding assay and an ELISA-based assay confirmed the high binding affinity and selectivity of the PTHTRWA peptide towards A549 cells (human adenocarcinomic alveolar basal epithelial cells). Based on these studies, we decided to introduce this heptapeptide onto the surface of extracellular vesicles and test their application potential in relation to cancerous human cells (A549) and human normal bronchial epithelial cells (BEAS-2B). In our previous work^23^ we showed that EVs functionalized with PTHTRWA and loaded with superparamagnetic iron oxide nanoparticle cargos reach the growing tumor when dosed intravenously in NUDE Balb/c mice bearing A549 cancer. The studies also showed that decoration of EVs with PTHTRWA resulted in high affinity and permanent binding of such conjugate to the membrane of cancer cells and more pronounced uptakes of PTHTRWA-EVs in cancerous A549 cells than noncancerous BEAS-2B cells. Identification of the cancer cell membrane receptor to which the heptapeptide binds will significantly accelerate its potential implementation in clinical practice.

In this work we examine the interactions between extracellular vesicles isolated from human lung cancer A549 cells and functionalized with a heptapeptide navigation ligand (PTHTRWA) and the integrins *α*5*β*1 and *α*2*β*1, belonging to the RGD-binding and collagen receptor families, respectively. In the recent study, the heptapeptide PTHTRWA was identified as a highly effective targeting ligand for engineering extracellular vesicles (EVs) toward lung cancer cells^23^. When conjugated through its C-terminus, PTHTRWA endowed EVs with markedly enhanced affinity and selective interaction with the membranes of A549 lung cancer cells compared to normal bronchial epithelial cells. Functionalized PTHTRWA-EVs also demonstrated effective tumor homing in preclinical MRI studies in mice and showed minimal cytotoxic or genotoxic effects, highlighting their potential as precision delivery vehicles. This targeted molecular recognition and structural adaptability of PTHTRWA make it a promising modular peptide for advancing EV-based personalized oncology therapeutics. Please note that integrin *α*5*β*1 is absent in normal lung tissue but is expressed in cancerous tissue^24^, and its overexpression is associated with enhanced malignancy, metastasis, and reduced patient survival^25^. In contrast, integrin *α*2*β*1 has been implicated in chemoresistance and may also contribute to reduced sensitivity to targeted therapies^26^. To elucidate the molecular basis of these interactions, we employed surface plasmon resonance (SPR) to characterize the binding PTHTRWA-labeled EVs to *α*2*β*1 and *α*5*β*1 integrins and to determine binding affinities and kinetic parameters associated with ligand engagement. Complementary molecular dynamics (MD) simulations were performed to model the PTHTRWA–*α*2*β*1 and PTHTRWA–*α*5*β*1 complexes, with the aim of describing residue-level interaction patterns and conformational preferences at the binding interface. Whereas MD simulations represent monovalent peptide-integrin interactions and enable mechanistic analysis of molecular recognition, SPR measurements reflect the multivalent avidity effects of PTHTRWA-decorated EVs. In this context, apparent binding strength is enhanced by rebinding events and cooperative interactions that are not captured in atomistic simulations. Taken together, these complementary approaches provide structural insight into integrin-ligand interactions and identify molecular determinants that may by exploited to enhance the specificity and therapeutic potential of EVs-based cancer-targeting strategies.

## Materials and methods

### Ligand-integrin *α*2*β*1: molecular dynamics simulation setup and parameters

MD simulations were performed to investigate the interactions and stability of heptapeptide-integrin complexes (PTHTRWA−*α*2*β*1, 6-AHA-PTHTRWA−*α*2*β*1, and PTHTRWA-3-APA−*α*2*β*1) in solution under conditions approximating physiological ionic strength. Simulations were conducted using the CHARMM force field^34^ in Discovery Studio v.2024 (BIOVIA). Each system was solvated in a rectangular TIP3P water box^35^, with the integrin positioned at least 10 Å from the box edges and immersed in a 0.15 M NaCl solution to replicate physiological ionic strength. Electrostatic interactions were treated using the particle mesh Ewald (PME) method^36^. The minimization process was initially conducted using molecular mechanics (MM) in three sequential stages. In the first stage, only the water molecules were optimized, while positional restraints were applied to the receptor. In the second stage, both the water molecules and the receptor side chains were subjected to optimization, with the receptor backbone remaining restrained. Each of these two stages involved two cycles of energy minimization: a total of 5,000 steps, beginning with steepest descent minimization and switching to the conjugate gradient method after 15,000 steps. In the final stage, the entire system was minimized without restraints over 5,000 steps, starting again with steepest descent and transitioning to conjugate gradient minimization after 10,000 steps. This process continued until the root-mean-square (RMS) gradient of the system fell below 0.1 kcal·mol^−1^·Å^−1^. All restraints applied in this study were weak, with a force constant of 2 kcal·mol^−1^·Å^−1^. Following energy minimization, the system was gradually heated from 50 K to 300 K over a period of 200 ps. Prior to the production phase, equilibration was performed by allowing the system to evolve freely until the average temperature and structural parameters stabilized, and the total energy reached convergence. The equilibration phase lasted 500 ps. Subsequently, the unrestrained system was subjected to a production run under periodic boundary conditions for 500 ns in the NPT ensemble, employing a Langevin thermostat. To investigate correlated motions, separate 500 ns molecular dynamics simulations were carried out for the ligand-free integrin, the ligand alone, and the integrin-ligand complex. Structural stability of the integrin during the simulations was evaluated based on equilibrated trajectories. Structural changes within the integrin-ligand complexes were characterized by calculating the root-mean-square deviation (RMSD) and root-mean-square fluctuation (RMSF) of backbone atoms. Single, extended MD trajectories were analyzed for each system. Although multiple independent replicates would improve statistical robustness, the convergence observed in RMSD and RMSF profiles together with consistent structural behavior across trajectories support the qualitative mechanistic interpretation presented here.

### Binding free energy calculations for ligand-*α*2*β*1 integrin interactions

To assess the relative stability and energetic characteristics of binding to integrin, the free energies of the integrin, the ligand, and the integrin–ligand complex were computed using the Molecular Mechanics/Poisson–Boltzmann Surface Area (MM/PBSA) method following MD simulations. MM/PBSA calculations were performed using the CHARMM-based implementation available in Discovery Studio v2024 (BIOVIA, Dassault Systèmes). Atomic coordinates for both the integrin and the ligand were extracted from a single MD^37–39^ trajectory obtained in the presence of explicit water molecules. The final 10 ns of each trajectory were selected for MM/PBSA analysis, as RMSD and RMSF plots indicated structural convergence within this equilibrated window. Prior to energy evaluation, each component of the complex (integrin, ligand, and complex) was subjected to energy minimization using a coupled gradient approach consisting of 10,000 steps, initiated with 1,000 steps of the steepest descent algorithm. A dielectric constant of 4 was assigned to the integrin (protein interior) and 80 to the solvent, following common practice in MM/PBSA to implicitly account for polarization and flexibility in the binding interface^37,38^. The choice of *ε* = 4 balances electrostatic screening within the binding site, while higher values (e.g., 8–20) may alter electrostatic contributions and shift absolute Δ*G* magnitudes. Minimization proceeded until the root-mean-square gradient of the system dropped below 0.001 kcal·mol^−1^·Å^−1^. The computed binding free energy (Δ*G*_bind_) incorporates both conformational flexibility of the integrin and dynamic positioning of the ligand within the binding site, thereby enhancing the reliability of the binding mode prediction. Δ*G*_bind_ was calculated as: Δ*G*_bind_ = *G*_complex_ – *G*_integrin_ – *G*_ligand_, where *G*_complex_ represents the total free energy of the integrin–ligand complex, and *G*_integrin_ and *G*_ligand_ are the corresponding free energies of the unbound integrin and ligand in solution, respectively. The MM/PBSA approach provides an approximate energetic description of single-molecule peptide-integrin interactions. These values are not directly comparable to SPR-derived apparent affinities for multivalent EVs binding, which are dominated by avidity and surface effects.

### Surface plasmon resonance studies

The SPR experiments were performed using a Biacore X100 system (Cytiva) on an CM7 sensor chip in which the gold surface is modified with carboxymethylated dextran. CM7 sensor chip is characterized by a high degree of carboxylation, providing high immobilization capacity. Dextran carboxyl groups were used to covalently immobilize the *α* integrin. For this purpose, the -COOH groups of dextran were activated using an EDC/NHS mixture (40 mM / 10 mM). An activated sensor chip was placed in the measuring chamber. The stabilization of the CM7 sensor chip was carried out in 1⋅PBS Gibco buffer (pH 7.2) with the addition of 1.0 mM CaCl_2_ and MgCl_2_. After reaching the stable baseline, the solution containing integrin (10 µg⋅mL^−1^) was injected on the sensor chip surface (contact time: 500 s; flow rate: 10 µL⋅min^− 1^). The integrin molecules were covalently attached to a dextran-modified sensor chip *via* an amide bond. In the next step, the interaction between integrin and PTHTRWA-EVs took place (contact time: 180 s; dissociation time: 600 s; flow rate: 30 µL⋅min^− 1^). The same sensor chip could be used for several experiments, provided that the surface was properly regenerated by removing PTHTRWA-EVs and captured integrins. We adopted a two-step regeneration protocol using: 10 mM glycine-HCl pH 1.5, and 50 mM NaOH regeneration solutions. These solutions were injected at a flow rate of 30 µL⋅min^–1^ for 30 s. All used solutions were prepared in 1⋅PBS Gibco buffer (pH 7.2) with the addition of 1.0 mM CaCl_2_ and MgCl_2_.

### Preparation of input data

**Fig. 1:**
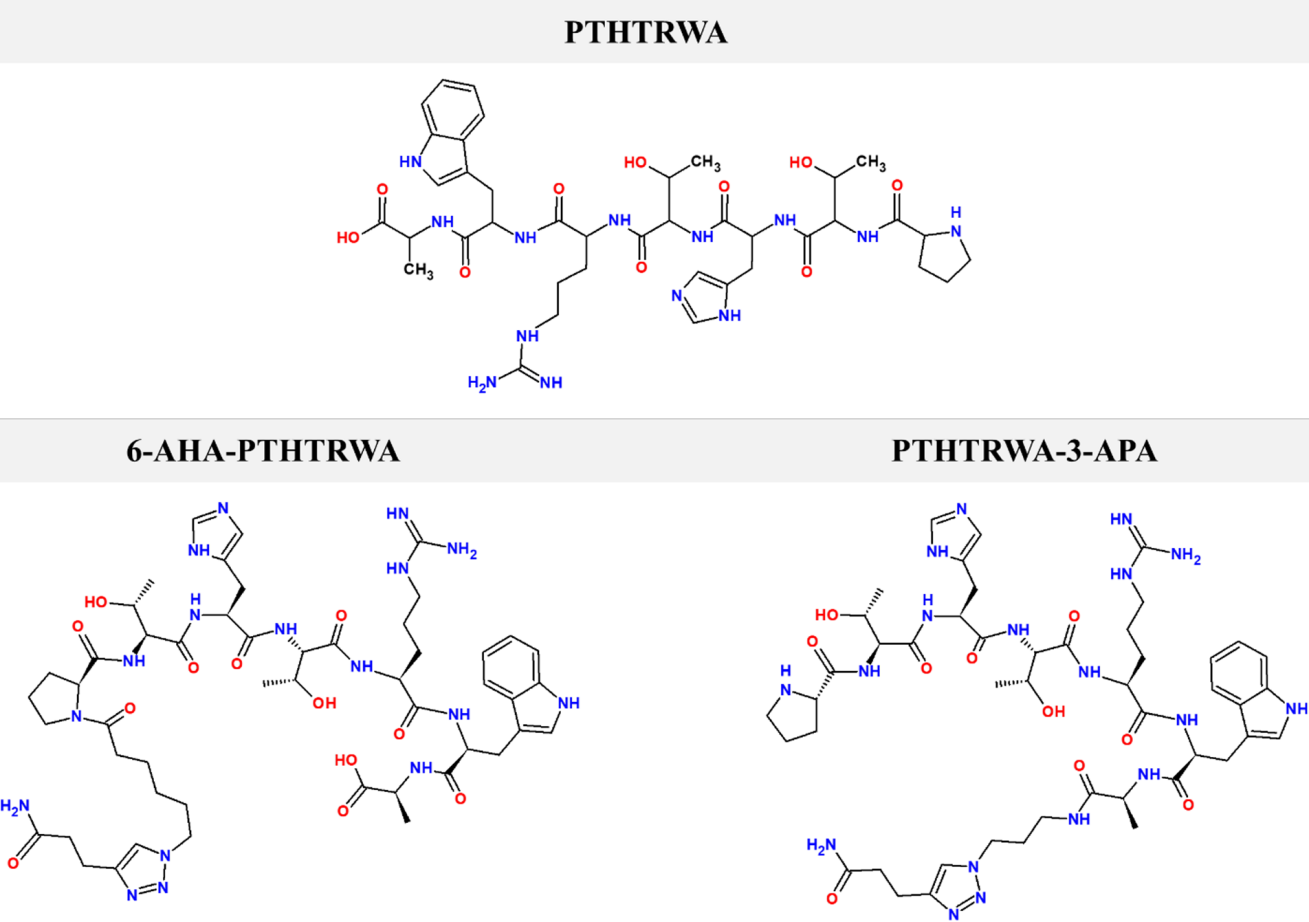
Chemical structures of heptapeptide PTHTRWA (H_2_N-Pro-Thr-His-Thr-Arg-Trp-Ala-OH), N-terminated heptapeptide (6-AHA-PTHTRWA), and C-terminated heptapeptide (PTHTRWA-3-APA).

The heptapeptide PTHTRWA (H_2_N-Pro-Thr-His-Thr-Arg-Trp-Ala-OH) and its N-terminally (6-AHA-PTHTRWA) and C-terminally (PTHTRWA-3-APA) modified analogs were synthesized and characterized in our laboratory as molecular probes for studying integrin–ligand interactions and conformational preferences of *α*2*β*1 and *α*5*β*1 integrins. The procedure for heptapeptide synthesis, as well as its comprehensive characterization, was described previously^23^. The structures of the synthesized PTHTRWA, 6-AHA-PTHTRWA, and PTHTRWA-3-APA, presented in Fig. 1, were optimized *via* density functional theory (DFT) at the B3LYP/6-311G(d, p) level using Gaussian 16^27^. Partial atomic charges were assigned through electrostatic potential fitting with the Breneman model^28^, ensuring consistency with the molecular electrostatic potential. Six ligand-bound complexes representing the open and closed conformations of the *α*2-I domain in human integrin *α*2*β*1, as well as three complexes in human integrin *α*5*β*1, were analyzed as described previously^23^. The three-dimensional structures of the *α*2-I domain were retrieved from the RCSB Protein Data Bank: the closed conformation (PDB ID: 1AOX, 2.1 Å resolution)^29^, the open conformation (PDB ID: 1DZI, 2.1 Å resolution)^30,31^, and *α*5*β*1 integrin (PDB ID: 3VI4, 2.9 Å resolution)^32^. The open conformation (1DZI) includes a triple-helical collagen fragment bound at the metal ion-dependent adhesion site (MIDAS) coordinated by a Co^2+^ ion, while the closed conformation (1AOX) contains a Mg^2+^ ion at the MIDAS site. For consistency, the Mg^2+^ ion in 1AOX was replaced with a Co^2+^ ion at the same coordinates. The Co^2+^ ion was used as a consistent structural proxy with the metal in the open conformation (1DZI), ensuring uniform coordination geometry across models without altering the overall coordination environment of the binding site. Crystallographic water molecules and original ligands were removed to define the ligand-binding site. Explicit hydrogen atoms were added, and ionizable residues were protonated to reflect physiological pH (7.4). Molecular docking simulations were conducted using the CDOCKER protocol within the Discovery Studio v2024 suite (BIOVIA, Dassault Systèmes)^33^. CDOCKER employs a CHARMm-based molecular dynamics docking approach with full ligand flexibility. The binding site was defined as a 20 Å spherical region centered on the MIDAS site, with a grid extension of 8.0 Å. For each ligand, 30 random conformations were generated through 10,000 molecular dynamics steps. Simulated annealing involved 2,000 heating steps at 700 K, followed by 5,000 cooling steps to 300 K. Final poses were refined using full-potential minimization and ranked by CDOCKER energies, supplemented by analysis of ligand-receptor interactions. The top-ranked pose for each ligand served as the starting point for subsequent molecular dynamics simulations. The computational model represents a minimal single-peptide interaction with the integrin binding site and does not explicitly account for multivalent presentation on extracellular vesicles used in the experimental assays.

## Results

### Interaction of the PTHTRWA motif with transmembrane integrins: focus on *α*2*β*1

**Fig. 2:**
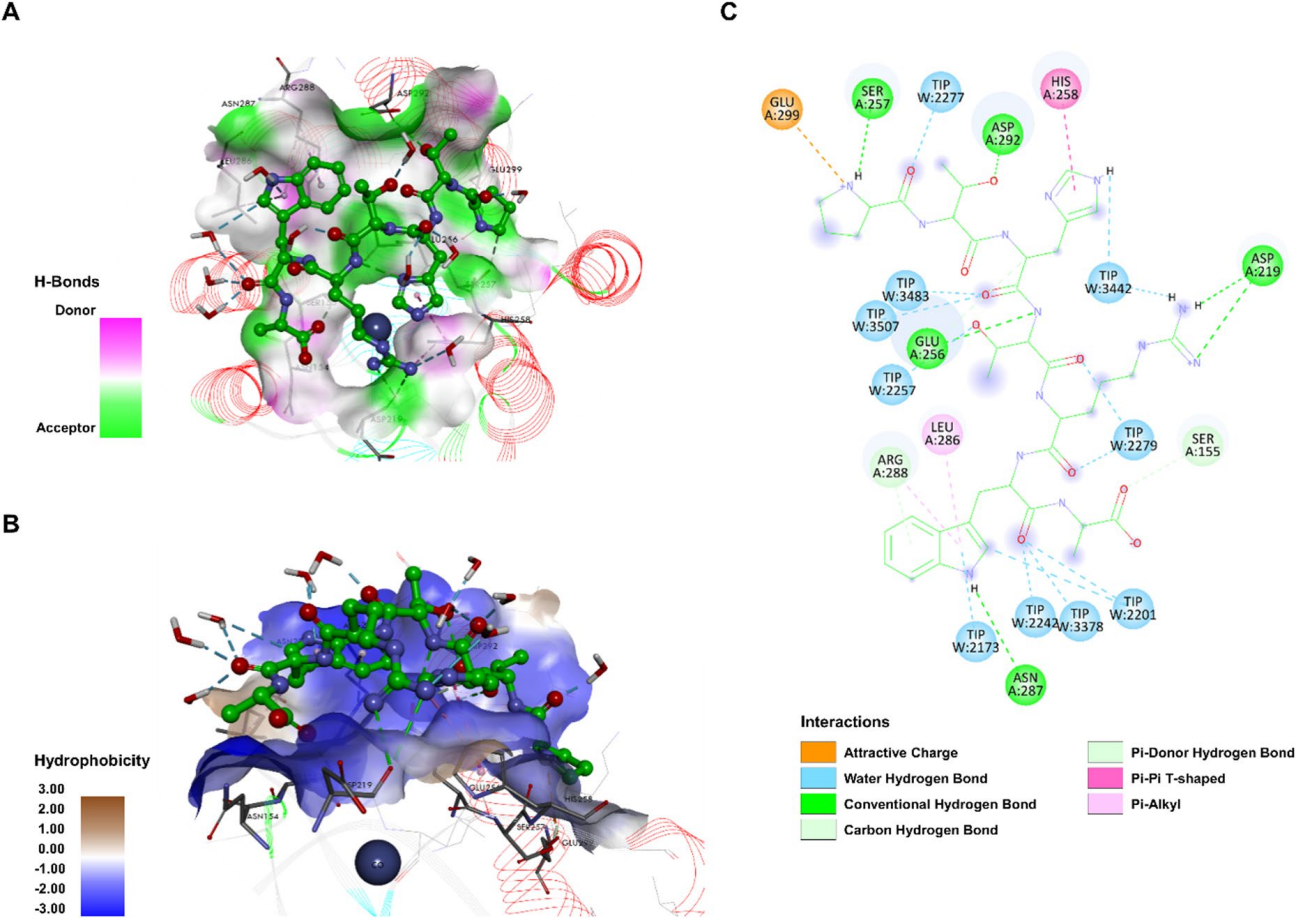
Binding mode of the PTHTRWA heptapeptide to the open I-domain of integrin *α*2*β*1, as revealed by MD simulations. (**A**) Hydrogen bond donor and acceptor surface of heptapeptide interacting with *α*2*β*1. Hydrogen bond donors are shown in pink regions, and acceptors in lime green. (**B**) Distribution of hydrophobic and hydrophilic residues surrounding the heptapeptide. Surface hydrophobicity is visualized by shaded colors: brown – indicates hydrophobic and blue indicates lipophilic regions. (**C**) 2D interaction map showing *α*2*β*1 residue interacting with the heptapeptide. Residues involved in hydrogen bonding are marked with green and cyan circles, hydrophobic interactions with pink circle, and electrostatic interactions with orange circle.

To investigate the structural determinants of PTHTRWA binding to *α*2*β*1 integrin, a computational model of the heptapeptide PTHTRWA docked onto the *α*2-I domain was generated. This model was based on the open crystal structure 1DZI and the closed structure 1AOX, incorporating the middle chain B and the trailing chain A of the triple helix (see Figure S1 in the Supplementary Information). The most important structural changes associated with the transition between the open and closed conformations include: (i) the unwinding of the RC helix (Gly-284–Tyr-285–Leu-286–Asn-287–Arg-288), followed by (ii) the formation of an additional turn in the R6 helix, and (iii) subsequent changes in coordination at the MIDAS site^30^. Based on these observations and previous research^40,41^, key interactions were identified for the efficient binding into the *α*2*β*1 integrin: (i) coordination with the Co^2+^ ion in the MIDAS loop (Ser-153, Ser-155, and Thr-221), (ii) hydrophobic contacts with His-258, Leu-286, and Leu-291, (iii) hydrogen bonding with Asn-154, Ser-257, Tyr-285, and Asn-287, and (iv) potential salt bridges interactions with Asp-219, and Glu-256. These interaction patterns were monitored throughout the simulations. The docking results indicate that PTHTRWA adopts a bent conformation compatible with the curvature of the integrin binding cavity. Water-mediated contacts also contributed to stabilizing the complex, consistent with the shallow nature of the binding interface between PTHTRWA and integrin (see Figure S2 in the Supplementary Information). The structure of the complex obtained from flexible docking with the *α*2*β*1 I-domain served as the starting configuration for MD simulations. In MM/PBSA analysis, the I-domain displayed more favorable interaction energies with the ligand in the open conformation (-14.84 kcal·mol^−1^ for 1DZI) compared with the closed conformation (-11.43 kcal·mol^−1^ for 1AOX). Although absolute binding energies derived from MM/PBSA are subject to methodological limitations, these values provide a qualitative indication of conformational preference for ligand binding. Consequently, open and closed conformations are often described in the literature as high-affinity (open-like) and low-affinity conformations. Both conformations coexist on the cell surface, although the closed state is generally more prevalent^42–45^. As shown in Fig. 2, PTHTRWA occupied the binding cavity of the open integrin *α*2*β*1 and was surrounded by the residues Asp-219, Asn-154, Ser-155, Leu-220, Gly-255, Ser-257, Glu-256, His-258, Gly-260, Leu-286, Asn-287, Arg-288, Asn-289, Leu-291, Asp-292, Asn-295, Leu-296, and Glu-299.

**Fig. 3:**
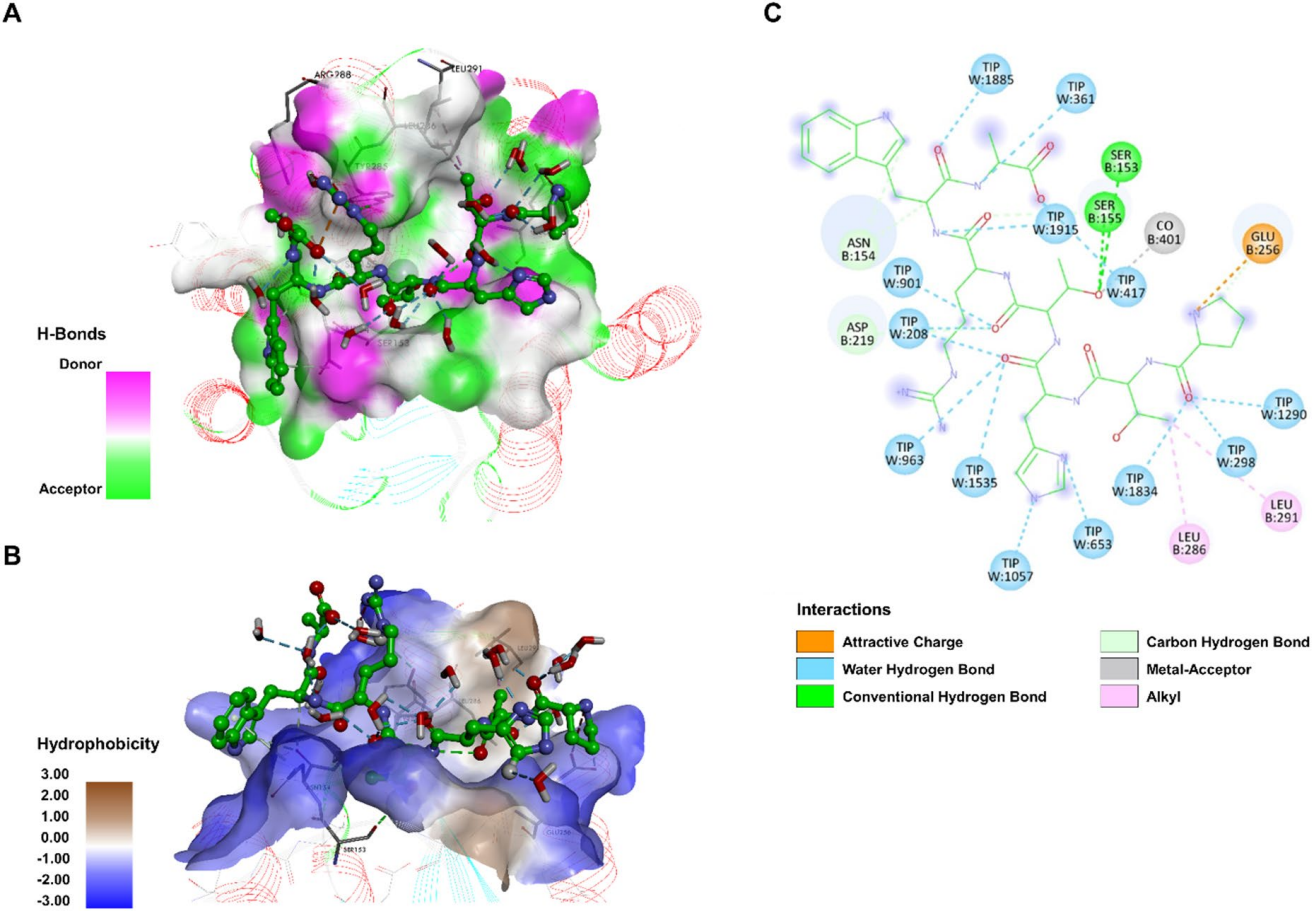
Binding mode of the PTHTRWA heptapeptide to the closed I-domain of integrin *α*2*β*1, as revealed by MD simulations. (**A**) Hydrogen bond donor and acceptor surface of heptapeptide interacting with *α*2*β*1. Hydrogen bond donors are shown in pink regions, and acceptors in lime green. (**B**) Distribution of hydrophobic and hydrophilic residues surrounding the heptapeptide. Surface hydrophobicity is visualized by shaded colors: brown – indicates hydrophobic and blue indicates lipophilic regions. (**C**) 2D interaction map showing *α*2*β*1 residue interacting with the heptapeptide. Residues involved in hydrogen bonding are marked with green and cyan circles, hydrophobic interactions with pink circle, and electrostatic interactions with orange circle.

The interaction mechanism between PTHTRWA and integrin involved a combination of hydrogen bonding and hydrophobic contacts, as shown in Fig. 2A and B. The simulations did not indicate direct hydrogen bonding between PTHTRWA and the Co^2+^ ion at the MIDAS site; instead, the peptide formed multiple hydrogen bonds within the binding pocket. Figure 2A presents the hydrogen bond surface area between PTHTRWA and the integrin binding interface, with residues acting as both donor or acceptor, indicating that hydrogen bonding extends along the cavity. The position of PTHTRWA peptide enables the formation of seven hydrogen bonds: Pro^PTHTRWA^ with Ser-257 (2.35 Å); Thr^PTHTRWA^ with Asp-292 (2.82 Å) and Glu-256 (2.32 Å); Arg^PTHTRWA^ with Asp-292 (2.03 Å and 2.46 Å); Trp^PTHTRWA^ with Asn-287 (2.80 Å); and Ala^PTHTRWA^ with Ser-155 (2.54 Å). Additionally, hydrophobic and electrostatic interactions were observed with the main chain of His-258, Leu-286, Arg-288, and Glu-299 at distances of 4.47, 5.41, 4.39, and 4.14 Å, respectively. Although salt bridges are frequently reported in integrin-ligand interactions, no salt bridge was detected in the PTHTRWA−*α*2*β*1 complex according to the applied geometric criterion (donor-acceptor distance ≤ 4.0 Å). In addition to direct protein contacts, the PTHTRWA heptapeptide formed stable water-mediated hydrogen bonds. The positioning of the peptide enabled water-bridged interactions involving His^PTHTRWA^, Thr^PTHTRWA^, Arg^PTHTRWA^, Trp^PTHTRWA^ and Pro^PTHTRWA^ residues, further contributing to stabilization of the binding interface. The results of the intermolecular interaction analysis indicate that the closed conformation of the I-domain of integrin *α*2*β*1 exhibits weaker interactions with the ligand compared to the open conformation (Fig. 3). The comparison of the open and closed conformations reveals that most regions involved in PTHTRWA binding on the surface of the I-domain of integrin *α*2*β*1 remain largely conserved during the transition between conformational states. Docking of the PTHTRWA heptapeptide indicated that one of one of the Thr^PTHTRWA^ residues was positioned in proximity to the Co^2+^ ion (2.11 Å), suggesting potential involvement in coordination geometry. Additionally, several characteristic interaction features were observed: Thr^PTHTRWA^ formed hydrophobic contacts with Leu-286 (5.40 Å) and Leu-291 (4.36 Å); the Pro^PTHTRWA^ fragment established both a hydrogen bond and an electrostatic interaction with Glu-256 (3.29 Å and 5.37 Å, respectively); and a second Thr^PTHTRWA^ residue formed hydrogen bonds with Ser-155 (3.03 Å), Ser-153 (3.21 Å) within the MIDAS motif, as well as with Asp-219 (2.63 Å) (Fig. 3C). The carbonyl and amino groups of Trp^PTHTRWA^, Ala^PTHTRWA^, Thr^PTHTRWA^, His^PTHTRWA^, and Pro^PTHTRWA^ also formed hydrogen bonds with water molecules, creating water bridges that extend the PTHTRWA heptapeptide within the receptor pocket and likely contribute to local stabilization of the binding interface.

### Stability of the *α*2*β*1- PTHTRWA complex assessed by MD simulation

**Fig. 4:**
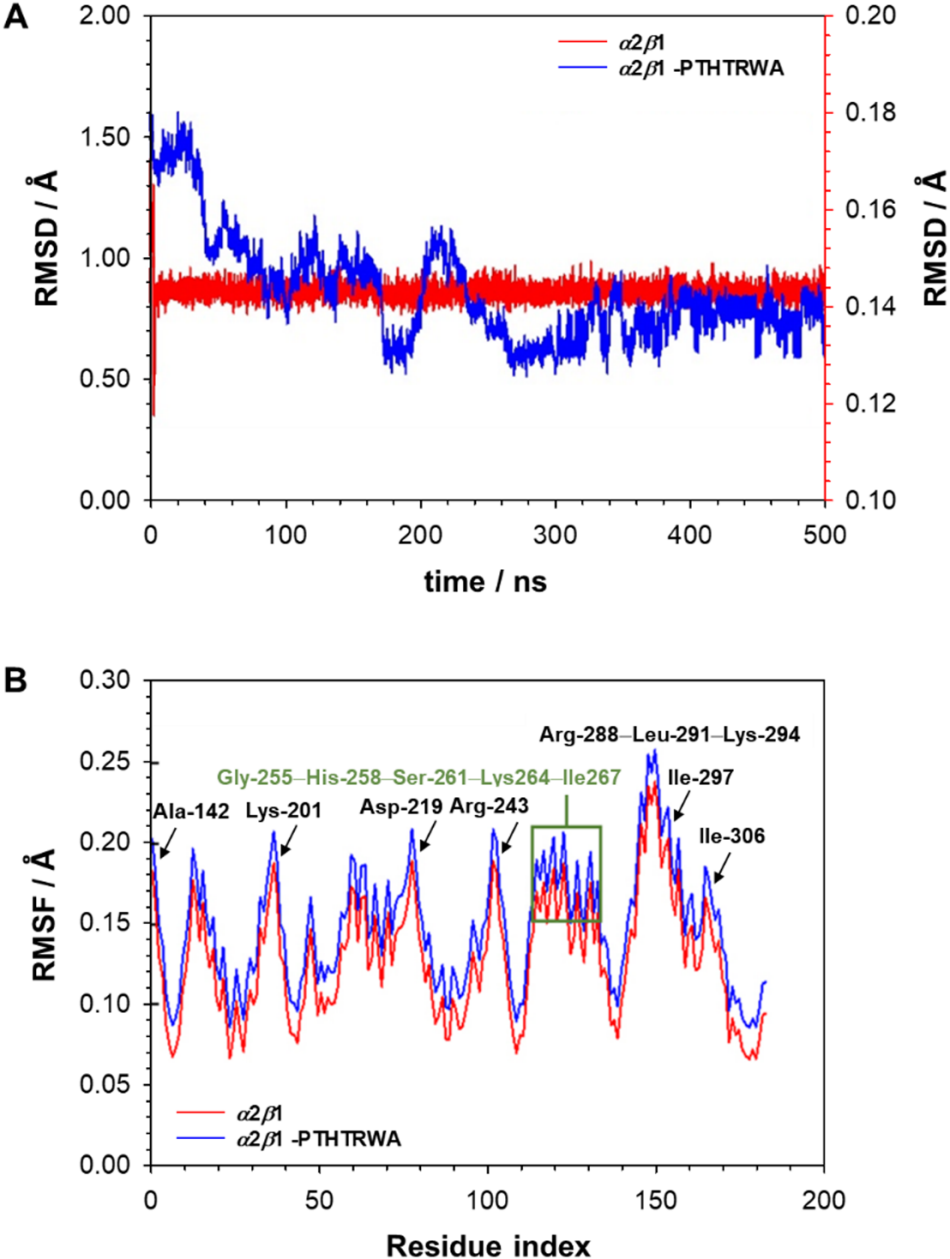
Molecular dynamics simulations of the open I-domain of integrin of *α*2*β*1 throughout 500 ns. (**A**) The RMSD plot of *α*2*β*1 skeleton with and without ligand PTHTRWA. (**B**) The RMSF values for amino acid residues with and without ligand PTHTRWA.

To evaluate the dynamic stability and conformational behavior of the *α*2*β*1–PTHTRWA complex, an all-atom MD simulation was performed for 500 ns under physiological temperature at 310 K. The simulation focused specifically on the open I-domain of the *α*2*β*1 integrin, which represents a ligand-accessible structural state relevant to integrin-ligand interactions. No extended MD production runs were performed starting from the ligand-bound closed conformation, as docking and equilibration indicated unstable or transient MIDAS coordination in this state, consistent with the low-affinity nature of the closed *α*2-I domain^30^. RMSD/RMSF analysis was therefore focused on the open ligand-bound state (Fig. 4), which represents the structurally relevant conformation for PTHTRWA binding. Key structural descriptors, including the root-mean-square deviation and root-mean-square fluctuation, were calculated to assess conformational stability throughout the simulation. As shown in Fig. 4A, the RMSD trajectory of the ligand-free I-domain remained consistently low, with minor fluctuations centered around 0.14 Å relative to the starting structure. These minimal deviations indicate structural stability of the open I-domain in the absence of the ligand. In contrast, the RMSD profile of the PTHTRWA-bound complex exhibited more pronounced structural deviations during the first half of the simulation, stabilizing after approximately 250 ns. Following this equilibration period, the system maintained an average RMSD of ~ 0.8 Å, suggesting that ligand binding is associated with initial conformational rearrangements within the binding cavity. The complex subsequently reached a stable conformational ensemble, consistent with stable accommodation of the PTHTRWA peptide within the open *α*2*β*1 in its open state.

To further characterize local residue-level flexibility, RMSF values were computed for all residues of the I-domain over the 500 ns trajectory (Fig. 4B). Most residues in proximity to the ligand-binding region exhibited low RMSF values (< 0.25 Å), indicating a well-constrained and structurally stable binding interface. The average RMSF for the complex ranged from 0.06 Å to 0.2 Å, reflecting moderate flexibility typical of native protein dynamics. The highest RMSF values were observed for Asn-289, Ala-290, and Asp-292, whose side chains are solvent-exposed and oriented away from the binding site, suggesting that their fluctuations are related to surface dynamics rather than direct ligand interactions. No significant destabilization or large-scale backbone rearrangements were detected within the ligand-binding region throughout the simulation. Collectively, the MD results indicate that PTHTRWA forms a stable complex with the open I-domain of *α*2*β*1 integrin while maintaining the overall structural integrity of the receptor. The stabilization observed after the initial adaptation phase supports further exploration of this heptapeptide as a potential targeting motif in EVs-based strategies.

### Impact of C- and N-terminal modifications of PTHTRWA on its binding interactions with integrin *α*2*β*1 and affinity toward integrin *α*5*β*1

**Fig. 5:**
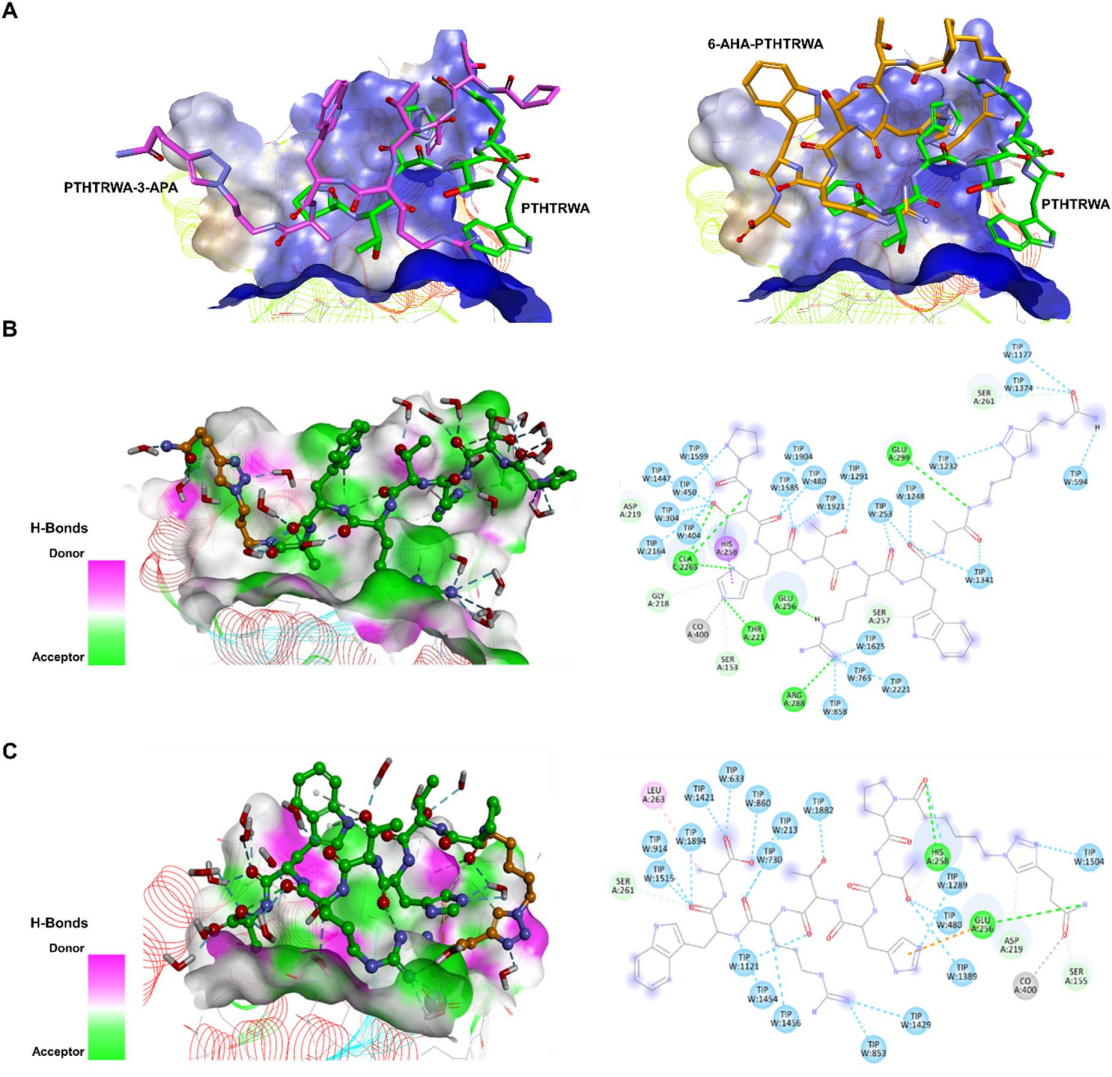
Predicted binding modes of N-terminally (6-AHA-PTHTRWA) and C-terminally (PTHTRWA-3-APA) modified heptapeptides within the *α*2*β*1 integrin binding pocket, as revealed by molecular dynamics (MD) simulations. (**A**) Structural superposition of the unmodified PTHTRWA (carbon atoms in green), 6-AHA-PTHTRWA (carbon atoms in orange), and PTHTRWA-3-APA (carbon atoms in pink), highlighting differences in binding orientations. (**B**) Hydrogen bonding surface and detailed interaction network between PTHTRWA-3-APA and the *α*2*β*1 integrin. (**C**) Hydrogen bonding surface and molecular contacts between 6-AHA-PTHTRWA and the *α*2*β*1 integrin. Hydrogen bond donors are visualized in pink, while acceptor regions are shown in lime green.

To explore structural factors that may contribute to the experimentally observed binding trends of EVs functionalized with the targeting heptapeptide PTHTRWA toward human lung cancer cells and tissues, we investigated how N- and C-terminal modifications of PTHTRWA influence its binding to the open I-domain of the *α*2*β*1 integrin. This approach builds on our previous studies^23^ where binding selectivity toward *α*5*β*1 integrin was analyzed. The present study provides structural insights into sequence-dependent determinants governing PTHTRWA interactions with *α*2*β*1 integrin. Comparative analysis of *α*2*β*1 and *α*5*β*1 integrin structures, supported by molecular simulations, revealed that PTHTRWA and its analogs − 6-AHA-PTHTRWA (N-terminal modification) and PTHTRWA-3-APA (C-terminal modification) - exhibit reduced stability in complex with *α*2*β*1 compared to their interactions with *α*5*β*1. Molecular dynamics simulations of the modified ligands in complex with *α*2*β*1 integrin demonstrated notable conformational flexibility, particularly within the I-domain binding region (Fig. 5). Trajectory analysis revealed ligand-dependent geometric preferences, indicative of dynamic adaptation to the receptor surface. As shown in Figs. 5A-C, both modified and unmodified forms of PTHTRWA preferentially localize on the surface of the open I-domain of *α*2*β*1 integrin. Key residue-level interactions identified during MD simulations are summarized in Table S1 in the Supplementary Information. Although the terminal modifications appear partially solvent-exposed in static structural models, MD trajectories indicate that they modulate local interaction networks and transient contacts at the interface. These observations suggest that comparable experimental Δ*G* values do not necessarily imply identical binding modes, as distinct conformational ensembles may display similar thermodynamic profiles. Notably, the presence of 3-azido-1-propanamine (3-APA) and 6-azidohexanoic acid (6-AHA) alters the binding mode, with these functional groups occupying distinct positions within the *α*2*β*1 binding pocket compared with their orientation in the *α*5*β*1 integrin complex. To quantitatively describe ligand-receptor interactions, binding free energies (Δ*G*_bind_) were estimated using the MM/PBSA method. These values are summarized in Table 1, together with the experimental free energies derived from Gibbs adsorption isotherms (Δ*G*_exp_ = -*RT* ln*K*_D_). For PTHTRWA-3-APA, the experimentally determined dissociation constants (*K*_D_) were 42.5 pM for *α*2*β*1 and 23.4 pM for *α*5*β*1, corresponding to Gibbs free energies of -14.13 kcal·mol^−1^ and − 14.49 kcal·mol^−1^, respectively, indicating a slightly stronger affinity toward *α*5*β*1 integrin. In contrast, MD simulations predicted interaction energies of -12.60 kcal·mol^−1^ for the PTHTRWA-3-APA with *α*2*β*1 complex and − 24.90 kcal·mol^−1^ for *α*5*β*1. For the 6-AHA-PTHTRWA, the computed binding free energy in complex with *α*2*β*1 was − 10.35 kcal·mol^−1^. These observations highlight differences between computational estimates and experimental measurements. For *α*2*β*1, the simulated binding free energy was 1.53 kcal·mol^−1^ less favorable than the experimental value - within the typical uncertainty of MM/PBSA calculations. In contrast, the *α*5*β*1 MD simulations substantially overestimated the interaction energy, indicating limitations of the simplified computational model. Such discrepancies may arise from differences between crystallographic conformations and their dynamic solvated states, as well as from simplifications inherent to single-peptide simulations that do not account for multivalent EVs presentation. Energetic comparisons between analogs show that PTHTRWA-3-APA exhibits more favorable interaction energy with *α2β*1 (Δ*G*_bind_ = -12.60 kcal·mol^−1^) than 6-AHA-PTHTRWA (Δ*G*_bind_ = -10.35 kcal·mol^−1^), suggesting that N-terminal modifications may influence interaction patterns within the *α2β*1 binding pocket. Conversely, experimental data for *α*5*β*1 integrin indicate higher affinity of PTHTRWA-3-APA for this receptor isoform, highlighting the receptor-specific differences in ligand recognition. Variations in binding behavior are likely governed by structural differences between integrin subtype, including binding site topology and electrostatic surface potential, which influence ligand orientation and interaction networks. In integrin *α*2*β*1, ligand binding is primarily localized within the *α*2-I domain pocket, involving residues such as Ser153, Thr221, Asp219, Glu256, and Leu286. In contrast, interactions in *α*5*β*1 integrin extend across the *β*-propeller domain and the specificity-determining loop, resulting in a more surface-exposed interface.

**Table 1:** Theoretical and experimental free energy of heptapeptide PTHTRWA, N-terminated heptapeptide (6-AHA-PTHTRWA), and C-terminated heptapeptide (PTHTRWA-3-APA) binding to *α*2*β*1 and *α*5*β*1 integrins.

Complex	ΔG_bind_ [kcal·mol^−1^]	Δ*G*_exp_^a^ [kcal·mol^−1^]
*α*2*β*1-PTHTRWA	-14.84	−
*α*2*β*1-PTHTRWA-3APA	-12.60	-14.13
*α*2*β*1-6-AHA-PTHTRWA	-10.35	−
*α*5*β*1-PTHTRWA	-35.87	−
*α*5*β*1-PTHTRWA-3APA	-24.90	-14.49
*α*5*β*1-6-AHA-PTHTRWA	-20.10	−

^a^Δ*G*_exp_=-*RT*ln*K*_D_

To further characterize the molecular basis of the observed differences in binding, residue-level interactions between ligands and the I-domain of the *α*2*β*1 integrin were analyzed based on MD trajectories. As shown in Fig. 5B, residues Asp-219, Gly-218, Ser-153, Thr-221, Glu-256, Arg-288, Ser-257, Glu-299, Ser-261, together with the Co^2+^ ion, participate in interactions with PTHTRWA-3-APA. The binding mode is consistent with cation-mediated coordination at the MIDAS site, with stabilization associated with Co^2+^ (2.93 Å). Several hydrogen bonds contribute to the interaction network, including contacts between His^PTHTRWA-3-APA^ and the MIDAS residues Thr-221 (1.88 Å) and Ser-153 (2.73 Å), as well as Gly-218 (3.56 Å); Arg^PTHTRWA-3-APA^ and Glu-256 (1.87 Å) and Arg-288 (2.11 Å); Trp^PTHTRWA-3-APA^ and Ser-257 (3.56 Å); Thr^PTHTRWA-3-APA^ and Asp-219 (3.78 Å); and the 3-azido-1-propanamine substituent with Glu-299 (3.02 Å) and Ser-261 (2.43 Å). As observed for the parent peptide PTHTRWA peptide, PTHTRWA-3-APA also accepts a hydrogen bond from a structural water molecule, indicating a conserved hydration-mediated interaction. In contrast, MD simulations indicated that 6-AHA-PTHTRWA adopts a distinct binding conformation but does not efficiently occupy the *α*2*β*1 binding pocket (Fig. 5C). This analog formed fewer hydrogen bonds, including interactions between the 6-AHA substituent and His-258 (1.99 Å), Glu-256 (2.74 Å), Asp-219 (3.20 Å), and Ser-155 (2.65 Å), as well as coordination with Co^2+^ (1.85 Å). Moreover, hydrophobic and electrostatic contacts were identified between Ala^6-AHA-PTHTRWA^ and Leu (4.85 Å), as well as between His^6-AHA-PTHTRWA^ and Glu-256 (3.80 Å). The reduced number and altered geometry of interactions observed for 6-AHA-PTHTRWA are consistent with its less favorable interaction energy towards *α*2*β*1 integrin.

### SPR analysis

**Fig. 6:**
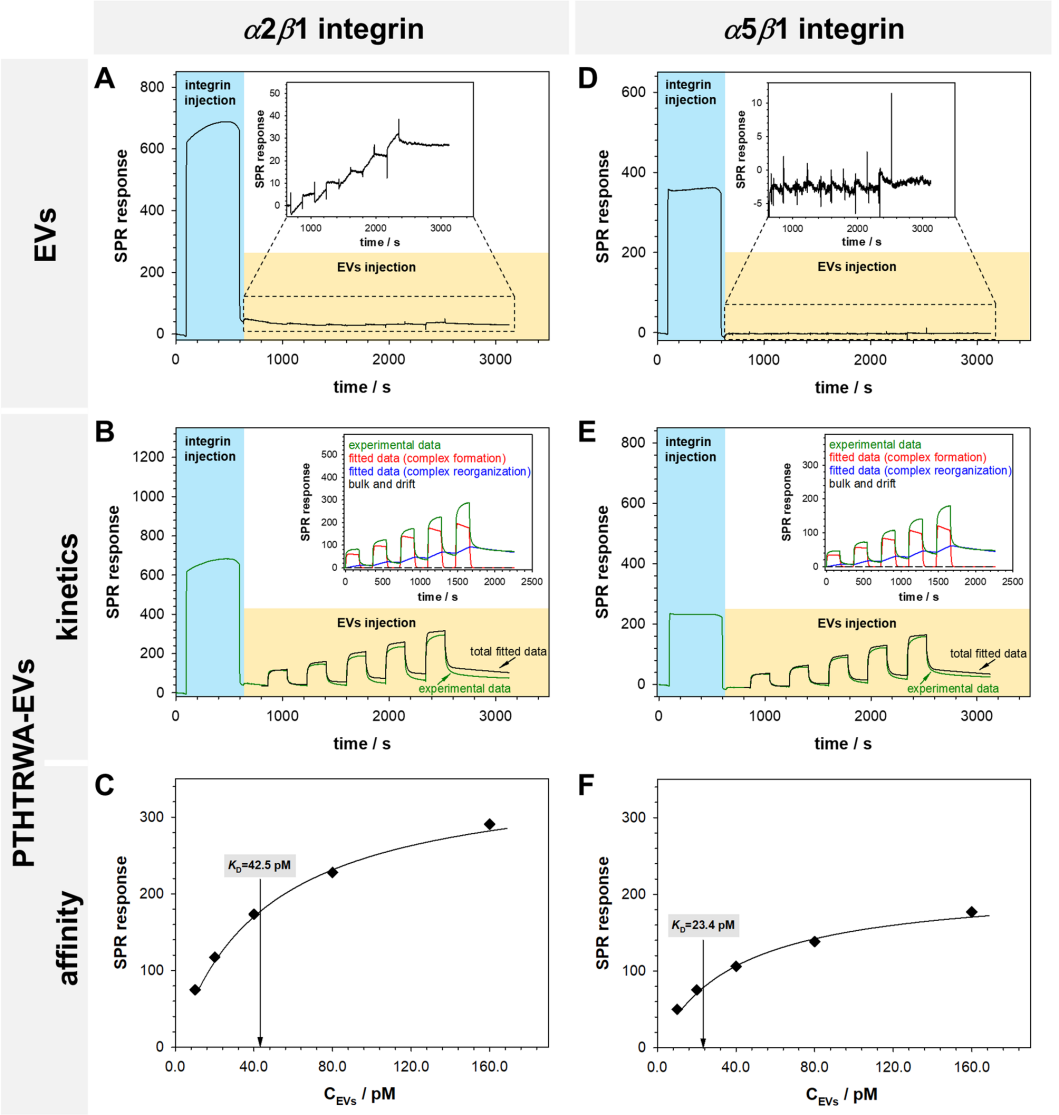
Sensorgrams recorded for the interactions of various concentrations of EVs (**A**, **D**) and PTHTRWA-EVs (**B**, **E**) with *α*2*β*1 and *α*5*β*1 integrins and their binding affinity plots (**C**, **F**). Insets: Enlarged area showing the interaction of EVs (A) and PTHTRWA-EVs (D) with integrins. Results of the fitting process, components of the curve fitted to experimental data (B, E). Experimental conditions: 1⋅PBS Gibco buffer (pH 7.2) with addition of 1.0 mM CaCl_2_ and MgCl_2_, *C*_*α*x*β*1_ = 10.0 µg·mL^–1^, *C*_EVs or PTHTRWA−EVs_: 10–160 pM.

The interactions of selected integrins (*α*2*β*1 and *α*5*β*1) and non-functionalized and functionalized with heptapeptide (PTHTRWA) EVs were monitored using SPR. The functionalization of extracellular vesicles can be reached through N-terminal or C-terminal amino acid. In our previous paper^23^ using human normal bronchial epithelial cells and human adenocarcinomic alveolar basal epithelial cells, we proved that EVs functionalized with the heptapeptide (PTHTRWA-EVs) through its C-terminus have a significantly higher affinity *versus* cancer cells. That is why in these studies we used only PTHTRWA-EVs. The typical SPR response curves recorded during immobilization of integrin molecules and their interaction with non-functionalized and functionalized EVs are shown in Fig. 6A-D.

The recorded sensorgrams evidently showed that the presence of heptapeptide on the surface of EVs promotes their interaction with integrins. An increase in the recorded signal was observed only for the stage of integrin immobilization, as well as their interaction with PTHTRWA-EVs. In the case of non-functionalized EVs the SPR signal confirming the interactions with integrins was not observed (insets in Fig. 6A and D). To get the kinetic parameters: association rate (*k*_a_), dissociation rate (*k*_d_), and equilibrium dissociation constant (*K*_D_), the two mathematical models (1 : 1 binding and two state reaction model) were fitted to the obtained experimental data. The chi-square values (for each fitting) for two state reaction model were at least two order lower than for 1 : 1 binding model. Therefore, the best model describing the interactions of integrins with PTHTRWA-EVs is a two-state reaction model. In the first step, the integrin-ligand complex (*α*x*β*1—PTHTRWA-EVs) is formed in 1 : 1 interaction mechanism, which is stabilized by a secondary process - conformational change of integrin from closed to open state. The determined kinetic parameters are presented in Table 2. The association rate constant defines the rate of complex formation, i.e. the number of *α*x*β*1—PTHTRWA-EVs complexes formed per second in a one molar solution of *α*x*β*1 and PTHTRWA-EVs. In turn, the dissociation rate constant describes the stability of the complex and determines how much of the complex has decayed in one second.

**Table 2:** Kinetic parameters of interactions between PTHTRWA-EVs and *α*2*β*1 and *α*5*β*1 integrins using a two-state reaction model.

*k*_a1_ [M^–1^·s^–1^]	*α*2*β*1—PTHTRWA-EVs	*α*5*β*1—PTHTRWA-EVs
	(7.59 ± 0.41)·10^6^	(6.61 ± 0.23)·10^6^
*k*_d1_ [s^–1^]	(6.05 ± 0.13)·10^–4^	(5.16 ± 0.18)·10^–4^
$\:\frac{{{k_{d1}}}}{{{k_{a1}}}}\: = \:{K_{D1}}\:[M]$	(7.97 ± 0.24)·10^–11^	(7.81 ± 0.01)·10^–11^
*k*_a2_ [s^–1^]	(1.75 ± 0.15)·10^–3^	(1.96 ± 0.31)·10^–3^
*k*_d2_ [s^–1^]	(8.40 ± 0.23)·10^–4^	(5.58 ± 0.28)·10^–4^
$\:\frac{{\mathrm{k}}_{\mathrm{d}\mathrm{2}}}{{\mathrm{k}}_{\mathrm{a}\mathrm{2}}}={\mathrm{K}}_{\mathrm{D2}}\:\mathrm{[}\mathrm{M}\mathrm{]}$	0.48 ± 0.03	0.28 ± 0.02
${K_D} = \:{K_{D1}} \cdot \:{K_{D2}}\:\:[pM]$	38.26 ± 3.61	22.23 ± 1.23
*K*_D_^*^ [pM] (kinetic model)	38.56 ± 2.77	17.3 ± 0.96
*K*_D_^*^ [pM] (affinity model)	42.50 ± 3.56	23.40 ± 1.08

^*^value determined by fitting of two-state reaction model.

MD simulations describe residue-level interaction patterns within a simplified single-peptide model, whereas SPR measurements capture the multivalent behavior of PTHTRWA-decorated EVs under experimental conditions. Accordingly, the two approaches provide complementary but not directly comparable information about integrin-ligand recognition.

## Discussion

Integrin *α*5*β*1 is a proangiogenic receptor that participates in vascular endothelial growth factor receptor (VEGFR) and angiopoietin-Tie signaling pathways, thereby contributing to tumor angiogenesis and progression^46^. Elevated expression of *α*5*β*1 integrin has been associated with tumor formation, metastasis, and resistance to chemotherapy and radiotherapy^47,48^. In non-small-cell lung cancer (NSCLC), *α*5*β*1 overexpression correlates with lymph node metastasis and reduced patient survival^24,49^. As a high-affinity fibronectin receptor, *α*5*β*1 enhances cancer cell adhesion and migration within fibronectin-rich tumors microenvironments^50^. Increased expression of *α*2, *α*5, and *β*1 integrin subunits has also been reported in tumor resistant to EGFR-targeted therapies, highlighting the clinical relevance of integrin-targeting strategies^51^.

**Fig. 7:**
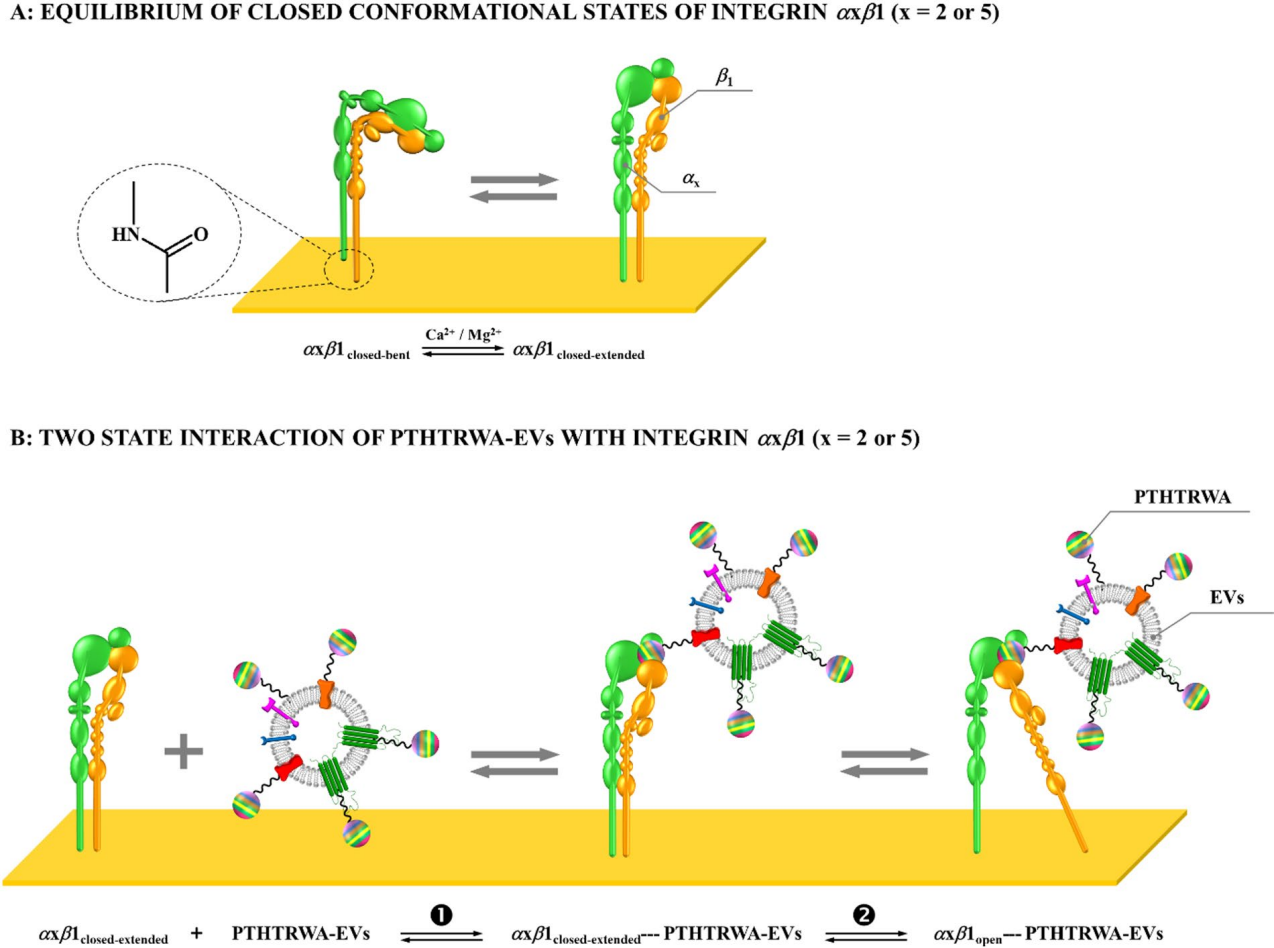
(**A**) Scheme of possible conformations of integrin in the closed state. (**B**) Scheme of interaction between PTHTRWA-EVs and *α*x*β*1 integrin.

Integrins undergo conformational transitions between bent or extended-closed and extended-open states, with ligand binding typically associated with the open, high-affinity structural arrangement. Experimental assays in this study were conducted in the presence of Ca^2+^ and Mg^2+^ ions, which support conformational transitions observed in structural studies^52^(see Fig. 7A). Kinetic parameters derived from SPR measurements indicated rapid initial association followed by a slower secondary phase, interpreted within the two-state kinetic model as a conformational transition (see Fig. 7B). Such behavior is consistent with previously reported integrin–ligand interactions, where electrostatic steering contributes to initial binding, while slower processes such as desolvation and local structural adjustments influence the overall kinetics^53,54^. The SPR-derived equilibrium dissociation constants showed comparable free energies of binding for *α*2*β*1 and *α*5*β*1 integrins, despite measurable differences in affinity. This apparent similarity can be rationalized by the logarithmic relationship between Gibbs free energy and the equilibrium constant (Δ*G*_exp_ = -*RT* ln*K*_D_), whereby even a twofold change in *K*_D_ corresponds to only ~ 0.4 kcal·mol^–1^ at room temperature. Consequently, relatively large variations in apparent affinity may translate into modest energetic differences.

Computational analyses provided complementary structural insight into peptide–integrin interactions at the residue level. Docking and molecular dynamics simulations indicate that PTHTRWA associates preferentially with the extended-open conformation of the integrin binding site, consistent with previous structural studies identifying this state as the high-affinity geometry. MM/PBSA calculations further support a conformational preference toward the open state, showing more favorable interaction energies compared with the closed conformation. Importantly, these simulations describe simplified single-peptide interactions and therefore provide qualitative insight into local binding determinants rather than quantitative predictions of multivalent EVs affinity. Despite relatively low sequence identity between *α*2 and *α*5 subunits (~ 25–30%), similar experimental Δ*G*_exp_ values suggest functional convergence arising from shared structural features, such as MIDAS coordination and conserved binding pocket architecture. Structural models indicate that N- and C-terminal modifications of PTHTRWA remain largely solvent-exposed and do not disrupt key interactions within the binding cavity. This observation is consistent with experimental SPR data showing comparable affinities for modified and unmodified peptides and suggests that flexible linkers used for EVs functionalization preserve integrin-targeting capability.

Detailed structural analysis indicates that PTHTRWA engages the integrin binding interface through a network of hydrogen bonds, hydrophobic contacts, and water-mediated interactions. MD simulations over 500 ns revealed stable conformational behavior of the *α*2*β*1—PTHTRWA complex, with local rearrangements confined primarily to the binding region and minimal perturbation of the global receptor structure. Comparative analysis of terminally modified analogs suggests that N-terminal modifications influence interaction patterns within the *α*2*β*1 binding pocket, whereas C-terminal modifications contribute more strongly to receptor selectivity, particularly toward *α*5*β*1 integrin. Differences between MM/PBSA-derived and experimental binding free energies highlight the limitations of simplified computational models. Binding free energies depend on methodological parameters such as dielectric constant and sampling window, and absolute Δ*G* values may vary accordingly. Furthermore, the computational systems represent isolated peptide–integrin complexes, whereas SPR experiments probe multivalent extracellular vesicles, where avidity, rebinding, and surface density effects significantly influence apparent affinity. Therefore, calculated Δ*G* values should be interpreted comparatively rather than as direct predictors of experimental binding strength. The largest discrepancies were observed for *α*5*β*1 integrin, where MM/PBSA substantially overestimated binding energies relative to experiment. This difference likely reflects structural and methodological factors, including the absence of an *α*-I domain in *α*5*β*1, differences in electrostatic surface properties, and increased entropic contributions associated with the *β*-propeller interface. Representative residue-level differences between the *α*2 and *α*5 binding interfaces are summarized in Table 3, highlighting variations in pocket polarity, MIDAS coordination, and electrostatic surface properties that may influence ligand recognition.

**Table 3:** Key differing residues in the ligand-binding sites of *α*2*β*1 and *α*5*β*1 integrins.

Region	α2 Subunit (I-domain)	*α*5 Subunit (*β*-propeller)	Potential structural implication
MIDAS-coordinating loop	Ser-153, Ser-155, Thr-221, Asp-219	Coordinated *via β*1 (Ser-123, Asp-227 equivalent)	Direct I-domain coordination in *α*2; distinct MIDAS organization in *α*5*β*1
Specificity-determining loop (SDL)	Hydrophobic (Leu-220, Ile-rich)	Polar (Asn, Gln-rich)	Differences in pocket polarity and solvation environment
Hydrophobic pocket	Leu-286, Arg-288, Glu-256	Variable loops with Phe, Tyr	May influence ligand insertion depth and solvent exposure
Electrostatic surface	Asn-154, Glu-256	Gln, Asn variants	Potential differences in desolvation contributions in simulations

Overall, MD simulations and SPR experiments provide complementary perspectives on integrin–ligand recognition. While SPR captures multivalent EVs behavior under experimental conditions, molecular simulations reveal structural features underlying peptide binding and conformational preferences at the atomic level. Together, these findings support further exploration of PTHTRWA-functionalized extracellular vesicles as integrin-targeting platforms and provide a structural framework for the rational design of modified ligands with improved selectivity.

## Supplementary Information

Below is the link to the electronic supplementary material.

Supplementary Material 1

Supplementary Material 2

## Data Availability

Data are available upon request from the corresponding authors.
