# Supplementary material for: Structural and mechanistic insights into α2β1 and α5β1 integrin targeting by bioengineered extracellular vesicles originating from lung cancer cells
Source: Sci Rep. 2026 Mar 27;16:10833. doi: 10.1038/s41598-026-46071-2 (PMC13039419; doi:10.1038/s41598-026-46071-2)
Supplement: Supplementary file 1 — Supplementary Material 1 [file 41598_2026_46071_MOESM1_ESM.docx]

**Supplementary Information**

**for**

**Structural and mechanistic insights into *α*2*β*1 and *α*5*β*1 integrin targeting by bioengineered extracellular vesicles originating from lung cancer cells**

Anna M. Nowicka^1*^, Teresa Żołek^2#^, Agata Kowalczyk^1#^ and Ireneusz P. Grudzinski^3*^

^1^University of Warsaw, Faculty of Chemistry, 1 Pasteura St, PL-02-093 Warsaw, Poland

^2^Department of Organic and Physical Chemistry, Faculty of Pharmacy, Medical University of Warsaw, 1 Banacha St, PL-02-097 Warsaw, Poland

^3^Department of Toxicology and Food Science, Faculty of Pharmacy, Medical University of Warsaw, 1 Banacha St, PL-02-097 Warsaw, Poland

* Correspondence to: [anowicka@chem.uw.edu.pl](mailto:anowicka@chem.uw.edu.pl); [ireneusz.grudzinski@wum.edu.pl](mailto:ireneusz.grudzinski@wum.edu.pl)

^#^ These authors contributed equally.


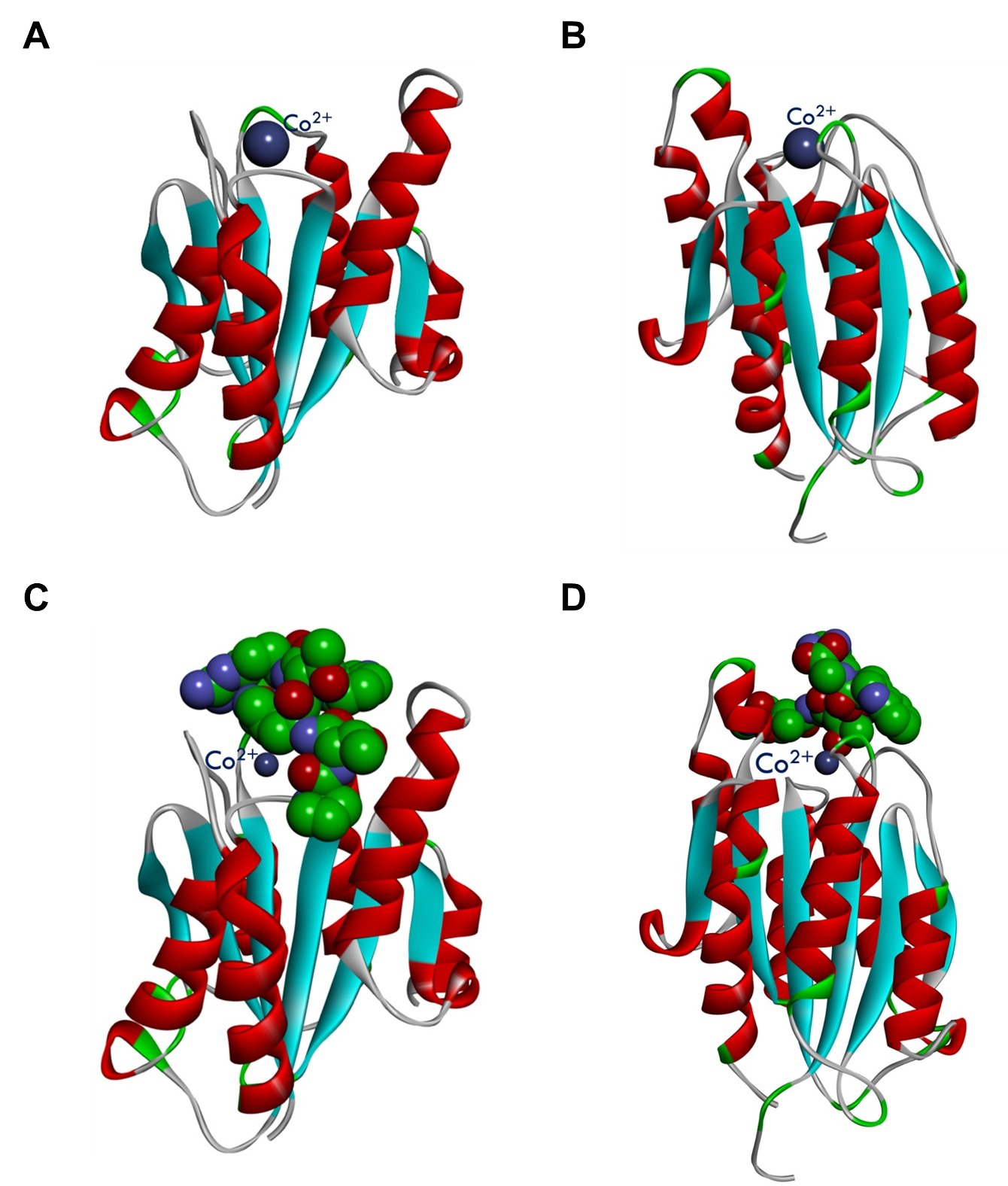


**Figure S1.** Crystallographic structures of the integrin *α*2*β*1 I-domain in the open (PDB: 1DZI) (**A**) and closed (PDB: 1AOX) (**B**) conformations. Structures of integrins in complex with the heptapeptide PTHTRWA after docking: the I-domain of integrin *α*2*β*1 in the open conformation (**C**), and the I-domain of integrin *α*2*β*1 in the closed conformation (**D**).

**
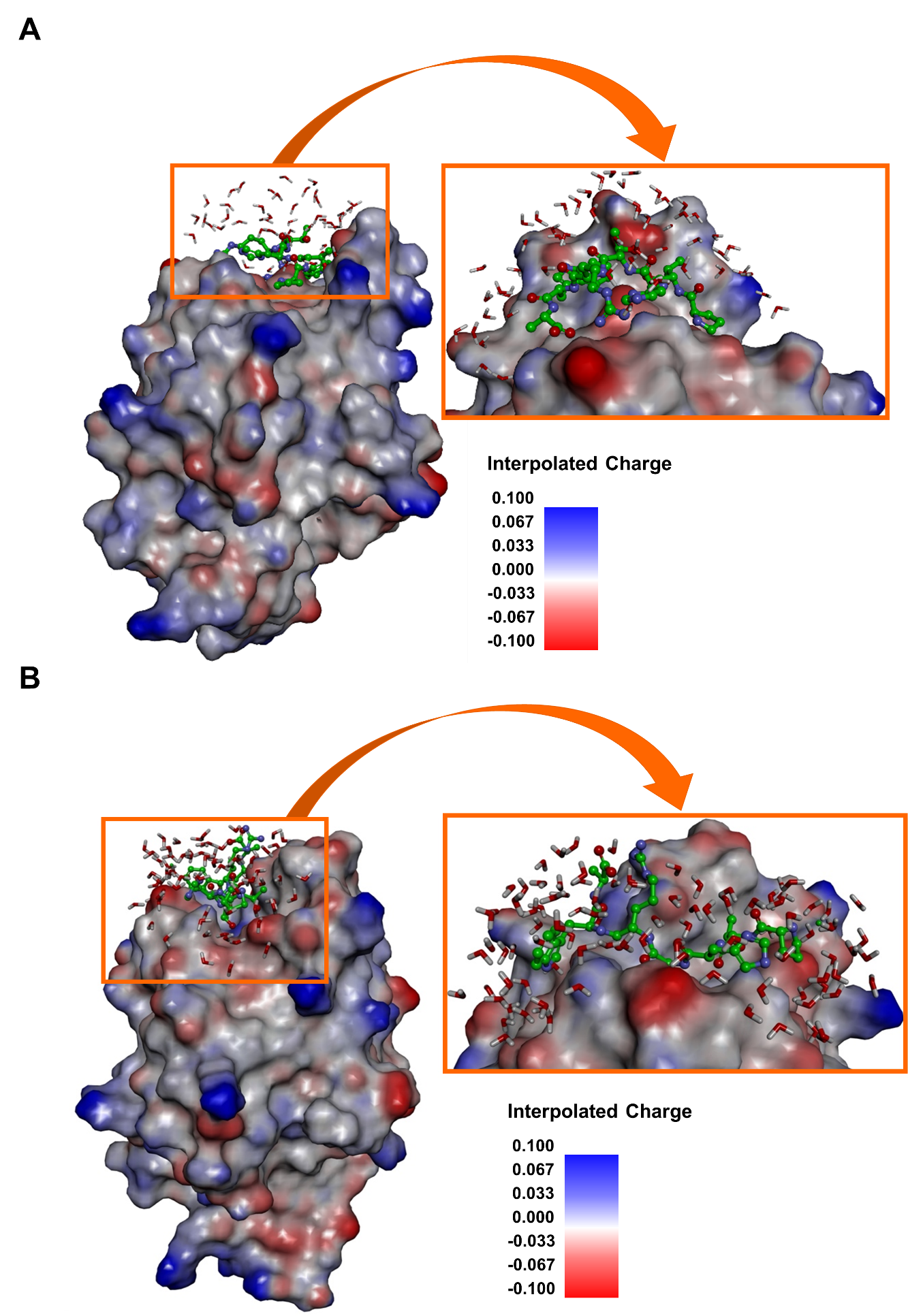
**

**Figure S2**. Location of water molecules and the heptapeptide PTHTRWA in the I-domain of integrin *α*2*β*1 in the open conformation (**A**) and in the closed conformation (**B**). The molecular electrostatic potential (MEP) map is shown on the surface, with interpolated coloring: blue for positive charge and red for negative charge.

**Table S1**. Summary of key residue-level interactions of PTHTRWA (and analogs) with *α*2*β*1 and *α*5*β*1 integrins from identified during MD simulations.

| **Integrin / conformation** | **Structural region or residues** | **Interaction type** | **Distance range (Å)** | **Occupancy (%)** | **Notes** |
| --- | --- | --- | --- | --- | --- |
| *α*2*β*1 **/** open (1DZI) | Ser153, Ser155, Thr221 | MIDAS metal coordination (Co^2+^) | 2.1-2.4 | 95-99 | stable coordination in active conformation |
| *α*2*β*1 **/** open | Asp219, Asn154 | hydrogen bonding | 2.8-3.2 | 82-94 | stabilizes ligand orientation |
| *α*2*β*1 **/** open | Leu220, Leu286, Arg288 | hydrophobic / *π*–cation contacts | <4.5 | 74-89 | deep pocket interactions |
| *α*2*β*1 **/** open | Glu256 | hydrogen bond / electrostatic | 3.0-3.5 | 65-85 | additional anchoring site |
| *α*2*β*1 **/** closed (1AOX) | Ser155, Thr221 | weak MIDAS coordination | >3.8 | 20-35 | reduced stability in inactive state |
| *α*5*β*1 (3VI4) | *β*1 MIDAS-equivalent residues | coordination and hydrogen bonding | 2.9-3.5 | 72-91 | main interaction interface |
| *α*5*β*1 | SDL loop (Asn/Gln-rich) | hydrogen bond / electrostatic | 3.1-3.8 | 58-82 | increased polarity of interface |
| *α*5*β*1 | *α*5 *β*-propeller loops | hydrophobic / van der Waals | <4.8 | 60–78 | contributes to receptor selectivity |
| Distances were calculated using standard geometric criteria (hydrogen bonds: donor–acceptor < 3.5 Å and angle > 120°; hydrophobic contacts:  C–C < 4.5 Å). Occupancy values correspond to the percentage of simulation frames (last 100 ns) in which a given interaction was observed. Comparable trends were obtained for PTHTRWA, PTHTRWA-3-APA and 6-AHA-PTHTRWA, with minor variations in side-chain contacts. | | | | | |
